# Weight Loss With Topiramate and Phentermine Combination Therapy in a Patient With Bardet-Biedl Syndrome

**DOI:** 10.1210/jcemcr/luaf164

**Published:** 2025-07-30

**Authors:** Doha Hassan, Mostafa Salama, Seema Kumar, Kalpana Muthusamy

**Affiliations:** Department of General Pediatrics and Adolescent Medicine, Mayo Clinic, Rochester, MN 55905, USA; Department of General Pediatrics and Adolescent Medicine, Mayo Clinic, Rochester, MN 55905, USA; Division of Pediatric Endocrinology, Department of Pediatric and Adolescent Medicine, Mayo Clinic, Rochester, MN 55905, USA; Division of Endocrinology, Diabetes, Metabolism, and Nutrition, Mayo Clinic, Rochester, MN 55905, USA

**Keywords:** Bardet-Biedl syndrome, BBS, obesity, topiramate, phentermine, weight loss, metabolic profile

## Abstract

There is limited data on the effect of antiobesity medications in individuals with Bardet-Biedl syndrome (BBS). Here, we present a case of an adult with BBS who experienced significant weight loss following treatment with a combination of topiramate and phentermine. A 31-year-old female with BBS, with homozygous pathogenic variant in the *BBS2* gene, presented with obesity, dyslipidemia, and obstructive sleep apnea. At 29 years of age, her body mass index (BMI) was 43.3 kg/m^2^ (reference range 18.5-24.9 kg/m^2^), and she was initiated on 50 mg topiramate daily for migraine prophylaxis. With lifestyle modifications, BMI dropped to 40.5 kg/m^2^. Subsequently, phentermine 30 mg daily was added. Eight months after adding phentermine, BMI significantly decreased to 31.5 kg/m² (27% decrease in BMI), with improvement in glycemic and lipid parameters. She developed paranoia due to a social stressor prompting a brief hold of phentermine and resumption at a lower dose. This case highlights the weight loss response with phentermine and topiramate combination therapy in a patient with BBS. Further research is needed to explore the efficacy and safety of conventional antiobesity medications in isolation, as sequential or combination therapy to setmelanotide, to most effectively impact weight and metabolic burden in patients with BBS.

## Introduction

Bardet-Biedl syndrome (BBS) is an autosomal recessive disorder characterized by a high prevalence of obesity, affecting approximately 72% to 86% of individuals [1]. Obesity and hyperphagia impose a substantial burden on patients and caregivers, significantly impacting quality of life [2]. Disruption of the hypothalamic melanocortin pathway, essential for regulating energy balance, leads to impaired signaling in patients with BBS, contributing to hyperphagia, reduced energy expenditure, and early-onset obesity [2]. The contributions of these factors and other potential pathophysiologic mechanisms underlying hyperphagia and obesity remain to be fully understood.

Setmelanotide, a melanocortin 4 receptor agonist, reduces hunger and body weight in patients with BBS and is approved for the treatment of obesity in those ≥2 years [2-4]. The high cost of setmelanotide, variable access, and the need for daily injections are barriers for consideration and long-term use. There is a need for data on the effiacy and safety of other weight-loss medications for managing obesity in patients with BBS.

The combination of phentermine and topiramate has demonstrated effectiveness in managing obesity and is approved for use in individuals ≥ 12 years [5]. No studies have assessed the effects of phentermine, topiramate, or their combination for obesity management in individuals with BBS.

## Case Presentation

We present a 31-year-old Hispanic female with BBS, diagnosed at 19 years, with a history of obesity since childhood. Peak body mass index (BMI) was 45.05 kg/m² [reference range (RR) 18.5-24.9 kg/m^2^] at 23 years. Genetic testing revealed a homozygous pathogenic variant in the *BBS2* gene (NM_031885.3, c.717 + 1G > T, GT donor). Weight- and BBS-related comorbidities included dyslipidemia, gastroesophageal reflux disease, retinitis pigmentosa, polydactyly, obstructive sleep apnea requiring bilevel positive airway pressure, and chronic hypoxic respiratory failure, requiring nocturnal supplemental oxygen. She also had bronchiectasis and migraine. Pulmonary comorbidities led to lung transplant evaluation, and weight loss was recommended, with a goal of BMI < 35 kg/m² for eligibility and to optimize outcomes. Physical activity was limited by easy fatigability secondary to her pulmonary disease; therefore, she engaged in chair-based exercises. Family history was remarkable for a sibling with BBS, type 2 diabetes mellitus in both parents, and heart disease in her father.

## Diagnostic Assessment

Physical examination was remarkable for obesity, developmental delay, and evidence of bilateral extra toe amputations. Computed tomography abdomen and pelvis showed persistent mild dilation of the renal collecting systems bilaterally. Thyroid functions were consistent with subclinical hypothyroidism with TSH 6.11 mIU/mL (6.11 µIU/L) (RR 0.36-4.20 mIU/mL) (0.36-4.20 µIU/L) and free FT4 1.05 ng/dL (13.52 pmol/L) (RR 0.72-1.70 ng/dL) (9.27-21.88 pmol/L).

## Treatment

**Figure 1.**
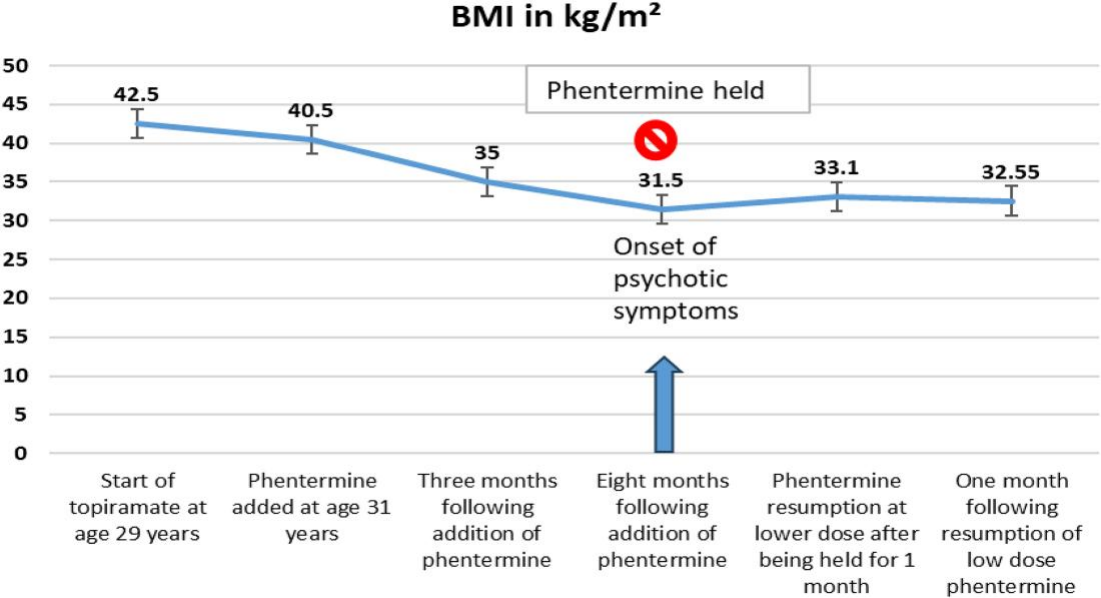
BMI changes secondary to phentermine and topiramate during the patient's clinical course: BMI trend demonstrating an overall decline in BMI from the initiation of topiramate, with a more pronounced decrease following the addition of phentermine.

Abbreviation: BMI, body mass index.

**Figure 2.**
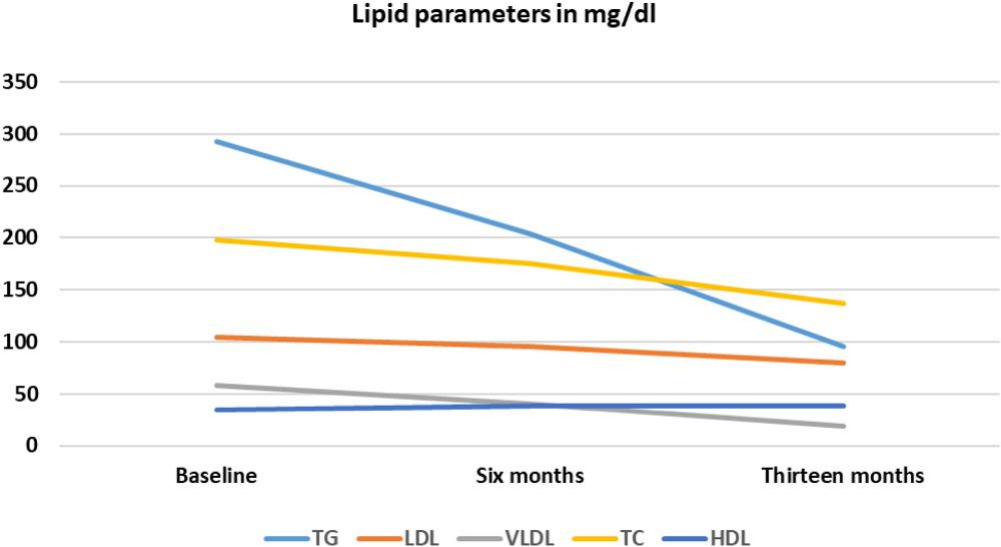
Lipid parameters' changes secondary to phentermine and topiramate during the patient's clinical course: overall trend of lipid parameters showing a significant reduction in triglycerides, a less pronounced decline in other lipid parameters, and an improvement in high-density lipoprotein levels, which remained stable over time. These trends were observed from the initiation of topiramate, through the addition of phentermine, to the most recent follow-up.

At age 29, she was initiated on topiramate 25 mg once daily, subsequently increased to 50 mg once daily, for migraine prophylaxis. Her baseline BMI was 42.5 kg/m² (Fig. 1). A baseline metabolic profile demonstrated hemoglobin A1c 5.5% (37 mmol/mol) (RR <5.7%) (<39 mmol/mol), fasting blood glucose (FBG) 101 mg/dL (5.61 mmol/L) (RR 70-100 mg/dL) (3.89-5.55 mmol/L), triglycerides (TG) 292 mg/dL (3.30 mmol/L) (RR 30-150 mg/dL) (0.34-1.70 mmol/L), very low density lipoprotein (VLDL) 58.4 mg/dL (1.51 mmol/L) (RR 8-30 mg/dL) (0.21-0.78 mmol/L), low-density lipoprotein (LDL) 104 mg/dL (2.69 mmol/L) (RR 70-100 mg/dL) (1.81-2.59 mmol/L), total cholesterol (TC) 198 mg/dL (5.13 mmol/L) (RR < 200 mg/dL) (<5.18 mmol/L), and high-density lipoprotein (HDL) 35 mg/dL(0.91 mmol/L) (RR 40-60 mg/dL) (1.04-1.55 mmol/L). (Fig. 2).

With lifestyle interventions, her BMI dropped to 40.5 kg/m². Setmelanotide was the drug of choice but was not covered by insurance. Injectable glucagon-like peptide-1 agonists due to their superior efficacy in terms of weight loss were also not covered by the patient's insurance, and therefore phentermine 30 mg daily was initiated as add-on therapy to 50 mg of topiramate. The 30 mg dose of phentermine was selected based on the severity of obesity (class III, BMI 42.5 kg/m²), the lack of data on the efficacy of combination therapy with phentermine and topiramate as formulated in Qsymia® (7.5/46 mg) in patients with BBS, and evidence demonstrating greater weight loss at 3 months with a 30 mg dose compared to a 15 mg dose [6].

## Outcome and Follow-up

After the addition of phentermine, the patient experienced a notable appetite-suppressive effect, reduced nighttime eating, sugar cravings, and stress-related eating. Additionally, she was able to increase the duration of exercise with improved endurance. She only reported constipation as a side effect.

Three months after adding phentermine, her BMI decreased to 34.96 kg/m². Repeat metabolic profile revealed FBG 70 mg/dL (3.89 mmol/L) with hemoglobin A1c 5.5% (37.5 mmol/mol), TG 204 mg/dL (2.31 mmol/L), VLDL 40.8 mg/dL (1.06 mmol/L), TC 175 mg/dL (4.53 mmol/L), HDL 38 mg/dL (0.985 mmol/L), and LDL 96 mg/dL (2.49 mmol/L). Eight months after adding phentermine, her BMI further declined by 27% to 31.5 kg/m².

The patient began experiencing paranoia 8 months after adding phentermine in the context of an abusive relationship, leading to significant emotional and psychological distress. She reported difficulty sleeping with frequent nightmares. Due to concerns that phentermine might exacerbate these symptoms, it was withheld for 1 month. During this period, she was no longer in the abusive relationship, and her sleep and symptoms of paranoia improved, although did not completely resolve.

After discontinuing phentermine, the patient noticed an increase in hunger. Nadir BMI was 31.5 kg/m² (Fig. 1). She experienced weight gain to BMI 33.1 kg/m². Phentermine was reintroduced at a reduced dose of 8 mg daily. She had also initiated trazodone 50 mg for insomnia. Referral was also provided for psychology and psychiatry to guide mental health support.

One month after restarting low-dose phentermine, she presented to the emergency department with worsening paranoid symptoms in the context of difficulty accessing outpatient psychological and psychiatric care. The significant psychological distress caused by the abusive relationship led to the development of flashbacks, nightmares, and suicidal ideation, resulting in hospitalization with a diagnosis of posttraumatic stress disorder.

During her hospitalization, she experienced a weight reduction with a BMI 32.55 kg/m². A repeat metabolic profile revealed FBG 75 mg/dL (4.17 mmol/L), TC 137 mg/dL (3.55 mmol/L), HDL 38 mg/dL (0.985 mmol/L), TG 96 mg/dL (1.09 mmol/L), LDL 80 mg/dL (2.07 mmol/L), and VLDL 19 mg/dL (0.49 mmol/L) (Fig. 2).

She was discharged after 4 days with complete resolution of her symptoms. At the time of this report, she is planning to undergo further inpatient mental health care, and follow-up weight management plan is yet to be finalized.

## Discussion

To the best of our knowledge, this is the first case report describing the effects of combination therapy with phentermine and topiramate for the treatment of obesity in a patient with BBS. Our patient experienced a substantial reduction in her BMI (27%) on a combination therapy of topiramate and phentermine, resulting in a significant reduction in BMI from 42.5 kg/m² to 31.5 kg/m² over a period of 10 months. Additionally, she had improvement in her FBG, TG, and VLDL levels.

No previous studies have assessed the efficacy or the degree of weight loss associated with phentermine, topiramate, or their combination for the treatment of obesity in patients with BBS.

In a clinical trial involving 64 women with obesity, participants were randomly assigned to receive either a placebo, continuous daily phentermine, or intermittent phentermine for 36 weeks, alongside a 1000-calorie/day diet. The mean weight loss was 10.58 lb (4.8 kg) in the placebo group, 26.90 lb (12.2 kg) in the continuous phentermine group, and 28.66 lb (13 kg) in the intermittent phentermine group. Participants on intermittent therapy experienced some weight regain during placebo periods, with weight loss resuming upon reintroduction of phentermine, indicating that its effectiveness is limited to periods of active use [7-9]. This is consistent with our patient's weight re-gain following the discontinuation of phentermine.

In a trial involving 118 patients with obesity (BMI 27-50 kg/m^2^), treatment with topiramate resulted in a mean weight loss of 5.9% in the 96 mg group and 6.5% in the 192 mg group after 28 weeks [10].

Extended-release phentermine and topiramate (Qsymia) is designed to provide a longer duration of action and an improved tolerability profile. In a phase III trial, patients receiving the higher dose of Qsymia (15/92 mg) achieved a mean weight loss of 14.4% after 56 weeks of treatment, compared to 6.5% in those receiving the lower dose (3.75/23 mg) [11]. The high cost of Qsymia and the lack of insurance approval limited its use in our patient.

Other alternative weight loss options for our patient included setmelanotide and glucagon-like peptide-1 receptor agonists. However, both were denied by her insurance with exclusion of weight loss medications from her insurance plan.

The side effect profile of topiramate includes cognitive impairment, memory deficits, visual disturbances, teratogenicity, and nephrolithiasis. Other adverse effects include neurological, gastrointestinal, and psychiatric symptoms [12].

Due to its structural similarity to amphetamines, phentermine has the potential for abuse. The most commonly reported side effects include dry mouth and insomnia [12]. Emerging evidence suggests that phentermine is not associated with an increased risk of major cardiovascular events and that its use, in the context of weight loss, may contribute to a reduction in blood pressure [12, 13]. Our patient continued to maintain normal blood pressure during treatment with topiramate and phentermine, along with the resolution of her migraine headaches.

Psychosis, which may manifest with symptoms such as paranoia, insomnia, irritability, and anxiety, is a well-documented adverse effect of phentermine, particularly in cases of longstanding use of high doses [14, 15].The literature describes a 25-year-old female who intermittently used diet pills containing phentermine 37.5 due to body image concerns. Following a social stressor, she began overdosing on phentermine, which resulted in psychotic symptoms within 3 to 4 weeks. Discontinuation of phentermine led to complete symptom resolution [16].

Our patient had been using phentermine for 8 months prior to the onset of paranoia, with no reported adverse effects aside from constipation. Psychotic symptoms occurred in the context of a social stressor, which may have been exacerbated by phentermine use. There is insufficient evidence to fully assess the long-term impact of phentermine on psychosis, the potential for certain individuals to be at higher risk, or the effects of gradual dose escalation with regular monitoring.

This case report highlights the substantial weight loss achieved with the combination of phentermine and topiramate in a patient with BBS. These findings underscore the need for further research to examine the long-term effects and safety profile of topiramate, phentermine, and their combination in patients with BBS. Exercising caution in individuals with underlying mental health conditions might be warranted.

## Learning Points

- The combination of phentermine and topiramate resulted in significant weight loss and significant improvement in metabolic profile in a patient with BBS.
- The effects of this combination therapy have not been formally studied in BBS, emphasizing the need for further research to explore the efficacy of these medications.
- Future studies to investigate the safety profile of topiramate, phentermine, or their combination in patients with BBS are warranted.

## Contributors

All authors made individual contributions to the authorship. K.M. was involved in the diagnosis and management of the patient, as well as reviewing and editing the manuscript. S.K. and M.S. contributed to reviewing and editing the manuscript. D.H. was responsible for reviewing the patient chart, collecting data, and writing and submitting the manuscript. All authors reviewed and approved the final draft.

## Data Availability

Original data generated and analyzed during this study are included in this published article or in the data repositories listed in references.
